# Tritium-Labeled Nanodiamonds as an Instrument to Analyze Bioprosthetic Valve Coatings: A Case of Using a Nanodiamond Containing Coating on a Pork Aorta

**DOI:** 10.3390/molecules29133078

**Published:** 2024-06-28

**Authors:** Maria G. Chernysheva, Tianyi Shen, Gennadii A. Badun, Ivan V. Mikheev, Ivan S. Chaschin, Yuriy M. Tsygankov, Dmitrii V. Britikov, Georgii A. Hugaev, Natalia P. Bakuleva

**Affiliations:** 1 Chemistry Department, M.V. Lomonosov Moscow State University, 3, bld. 1, Leninskie Gory, Moscow 119991, Russia; tianyi.shen@chemistry.msu.ru (T.S.); badunga@my.msu.ru (G.A.B.); mikheev.ivan@gmail.com (I.V.M.); 2 A. N. Nesmeyanov Institute of Organoelement Compounds, Russian Academy of Sciences, 28, bld. 1, Vavilova St., Moscow 119334, Russia; ivanchaschin@gmail.com; 3 A. N. Bakulev Scientific Center for Cardiovascular Surgery, 135, Rublevskoe Sh., Moscow 121552, Russia; tsigankov_yura@mail.ru (Y.M.T.); dbritikov@mail.ru (D.V.B.); george_kh87@mail.ru (G.A.H.); npbakuleva@yandex.ru (N.P.B.)

**Keywords:** xenogenic material coatings, nanodiamonds, heart valve prosthesis, radiotracer analysis, tritium-labeled compounds, pork aorta

## Abstract

Coatings with xenogenic materials, made of detonation nanodiamonds, provide additional strength and increase elasticity. A functionally developed surface of nanodiamonds makes it possible to apply antibiotics. Previous experiments show the stability of such coatings; however, studies on stability in the bloodstream and calcification of the material in natural conditions have yet to be conducted. Tritium-labeled nanodiamonds (negative and positive) were obtained by the tritium activation method and used to develop coatings for a pork aorta to analyze their stability in a pig’s bloodstream using a radiotracer technique. A chitosan layer was applied from a solution of carbonic acid under high-pressure conditions to prevent calcification. The obtained materials were used to prepare a porcine conduit, which was surgically stitched inside the pig’s aorta for four months. The aorta samples, including nanodiamond-coated and control samples, were analyzed for nanodiamond content and calcium, using the radiotracer and ICP-AES methods. A histological analysis of the materials was also performed. The obtained coatings illustrate a high in vivo stability and low levels of calcification for all types of nanodiamonds. Even though we did not use additional antibiotics in this case, the development of infection was not observed for negatively charged nanodiamonds, opening up prospects for their use in developing coatings.

## 1. Introduction

The importance of applying mechanical and biological prosthetic valves in modern cardiac surgery is substantiated by reviews and research papers [1,2,3,4,5]. There are several essential characteristics that a material should possess for prosthetic manufacturing. In terms of mechanical properties, the material should be soft enough to be opened by the blood flow, but firm enough to prevent blood from going backward. It also needs to eliminate calcific degeneration and blood coagulation. A calcific deposit is one of the causes [6,7,8] of narrowing of the valve opening, reducing blood flow through the valve. In the case of xenogenic heart valve prostheses treated with glutaraldehyde, calcification results from the attraction of host-plasma calcium ions to glutaraldehyde groups [7]. Masking the opposing groups of glutaraldehyde-modified surfaces is one of the ways to reduce calcium deposits. To this end, chitosan is a promising material to solve the problem [8,9,10,11].

Nanoparticles can be used to improve the mechanical characteristics of a biological material, while keeping it close to its natural quality. Our previous research shows that nanodiamonds are appropriate for this purpose [12,13,14]. Nanodiamonds produced by detonation techniques are inexpensive, biocompatible, nanoscaled, carbon-allotropic modifications characterized by functionally developed surfaces [15,16,17,18,19]. The advantage of detonation nanodiamonds is that they can be used for surface modification with drug molecules through chemical synthesis or adsorption [19,20,21,22,23,24]. Depending on the chemical treatment, nanodiamonds can have either positive or negative zeta potential in aqueous suspensions [25] and, therefore, can interact with different biologically active molecules. The unique properties of nanodiamonds make it possible to consider them applicable for drug delivery [22,26,27,28,29,30,31,32,33,34,35]. Note that nanodiamonds have an antibacterial effect [36,37,38] that can be improved by applying antibiotics [39]. The simplicity of the coating application and the results obtained regarding the improved characteristics allow us to consider nanodiamonds as a promising material for prosthetics [38,40,41]. In our previous studies, we used model systems, namely, the subcutaneous administration of the collagen material to rats, to determine the stability of the coating and predict the calcification of the material [12,14]. Let us highlight the novelty of the present research. First, it presents the stability analysis of a nanodiamond-containing coating in the bloodstream of a large animal. Second, all parameters, including the coating safety, calcification, and development of infections, have been investigated under the conditions of an actual product exploitation, while previously only models were used. Finally, the compositions of different surface functional groups for several types of nanodiamonds used as a basis of coating were analyzed.

To achieve this, a nanodiamond coating was applied to a pig’s aorta, which was surgically stitched to the animal for four months. The following three types of detonation nanodiamonds were used to prepare the coatings. The first was a nanodiamond powder, which, after sonication in water, has a positive zeta potential. The exposure of such nanodiamonds to air annealing results in the nanodiamonds’ carboxylation, changing the zeta potential to a negative one [42,43]. Nanodiamonds exposed to air annealing were the second type. Finally, nanodiamonds that are supplied by the manufacturer as an aqueous suspension and already have a negative zeta potential in this form were used to prepare coatings. All types of nanodiamonds were tritium-labeled by the tritium thermal activation method to enhance them with a radioactive property, making it possible to apply the radioactive tracer method to control the number of nanodiamonds in the coating after it had been exploited by the animal [44,45,46]. This method is based on the substitution of hydrogen with tritium by the reaction between the solid nanodiamond target and the tritium atoms that are generated on the surface of the tungsten filament at 1700–2000 K.

## 2. Results

### 2.1. Tritium Labeling of Nanodiamonds

In the present research, tritium labeling was performed on both positively and negatively charged nanodiamonds. The tritium thermal activation method allows a radioactive label to be introduced into both types of nanodiamonds without significant changes in their functional composition [44]. Since substitution occurs in all possible positions, including carboxyl-, amino-, and hydroxyl groups, the labeled product must be purified from labile tritium before using it as a tracer. The radioactivity of the labeled nanodiamonds used in this study is summarized in Table 1.

### 2.2. Analysis of Nanodiamond-Containing Coatings

Table 2 summarizes the number of nanodiamonds in the coatings directly after preparation and after their exploitation.

### 2.3. Histological Analysis Results

**Figure 1:**
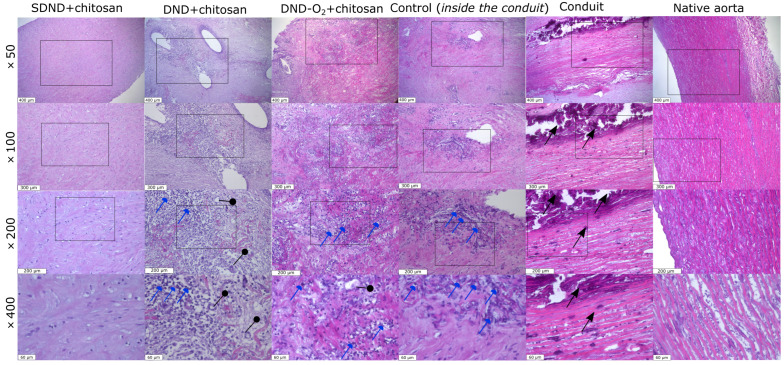
Sections of the tissue samples from various pork aorta samples. Columns from left to right: SDND+chitosan, DND+chitosan, DND-O2+chitosan, Control (inside the conduit), Conduit, Native aorta (×50, scale line—400 μm). Resolution scaling up of the sample images: ×100 (scale line—300 μm), ×200 (scale line—200 μm), ×400 (scale line—60 μm), stained with hematoxylin–eosin. Collagen and elastin fibers are colored pink, and the cells and their residues are colored blue. Black arrows indicate areas of calcification. Blue arrows represent areas of inflammation with lymphatic cells. Black pointers are sections of blood vessels formed during inflammation. Rectangles highlight the represented regions with high resolution.

Figure 1 shows sections of the tissue samples of various pork aorta samples.

The histological analysis shows that the wall of the native pork aorta (Figure 1, Native aorta) is represented by the inner-, middle-, and outer shells. The middle shell is built of many smooth muscle cells and pronounced elastic membranes, and there is no calcification or inflammation. This sample can be accepted as a positive control. For the carrier conduit (Figure 1, Conduit), it is possible to identify the middle shell of the aorta, mainly in the subintimal section, with the phenomenon of pronounced calcification. For the sample coated with DND+chitosan (Figure 1, DND+chitosan), it is possible to identify the phenomena of fibrosis and focal lymphohistiocytic infiltration with an admixture of giant multinucleated cells of foreign bodies, mainly in the area of holes from the suture material (chronic inactive inflammation). The control sample (Figure 1, Control (inside the conduit)) and the sample coated with DND-O2+chitosan (Figure 1, DND-O2+chitosan) are characterized by the phenomena of pronounced fibrosis and focal lymphohistiocytic infiltration (chronic inactive inflammation, less pronounced than in the case of DND+chitosan coating). On the contrary, the analysis results of the sample with the SDND+chitosan coating look very encouraging. Indeed, in this case, the fragment of the aortic wall shows no signs of fibrosis, calcification, or inflammation.

Purification results in a radioactivity decrease of 25–50%. Our previous experiments on preparing nanodiamond coating on collagen matrices show that boiling the material in concentrated nitric acid does not result in the formation of tritiated water leaving tritium at the precipitate phase after centrifugation [12]. This observation confirms the binding of tritium in CH bonds on the nanodiamond surface. Please note that the preparation of tritium-labeled nanodiamonds of high radioactivity was not the task of the present research because the sample was mixed with the corresponding non-radioactive nanodiamonds to prepare a suspension for aorta saturation. In the present study, we used tritium-labeled nanodiamonds to prepare a coating for the aorta and the experiment was conducted on a large animal for the first time.

Several additional controls were used during the ICP-AES and histological analysis implementation.

(1) A control (inside the conduit) (#4 in Figure 3a) using a sample of the pork aorta devitalized by a hybrid approach using a 1% solution of sodium dodecyl sulfate (SDS) and supercritical CO_2_ followed by coating with chitosan from carbonic acid at a pressure of 30 MPa [47]. This sample was sewn inside a donor pork aorta carrier [48]. 

(2) The donor pork aorta, inside of which the experimental coated samples were sewn, was devitalized by a hybrid approach using a 1% solution of SDS and supercritical CO_2_ (allograft) like a control sample, and which was installed inside the native aorta of the experimental animal. 

(3) The final sample was the native pork aorta, inside of which the allograft was installed (two samples taken near to and far away from the allograft). The content of calcium and other metals that can usually be determined in nanodiamonds as admixtures is summarized in Table 3.

The allograft was calcined to a similar degree, as outlined in Table 3. It was observed that the nanodiamond-containing coating did not increase calcium growth on the surface of the aorta. The presence of iron in these samples can be considered as a typical admixture in detonation nanodiamonds [49].

## 3. Discussion

In the present research, we have analyzed the stability and calcification of different types of aorta coating and the surface functional composition of detonation nanodiamonds and chitosan. The data obtained show the stability of the nanoparticles in the coating composition when being exploited by the pig for four months. The radiotracer method indicates the presence of nanodiamonds of all types in the coating. It was found that a significant number of nanoparticles remain on the surface of the aorta after the exploitation. The proportion of the residual amount was slightly higher for the negatively charged nanodiamonds than for the positively charged ones. Since a radioactive label remained at the nanodiamond phase after the decomposition of biological tissue in nitric acid, this allowed us to analyze the nitric acid solution for calcium content. This is an undoubted advantage of the developed technique because it allows us to obtain complete information about the material after its exploitation.

**Figure 2:**
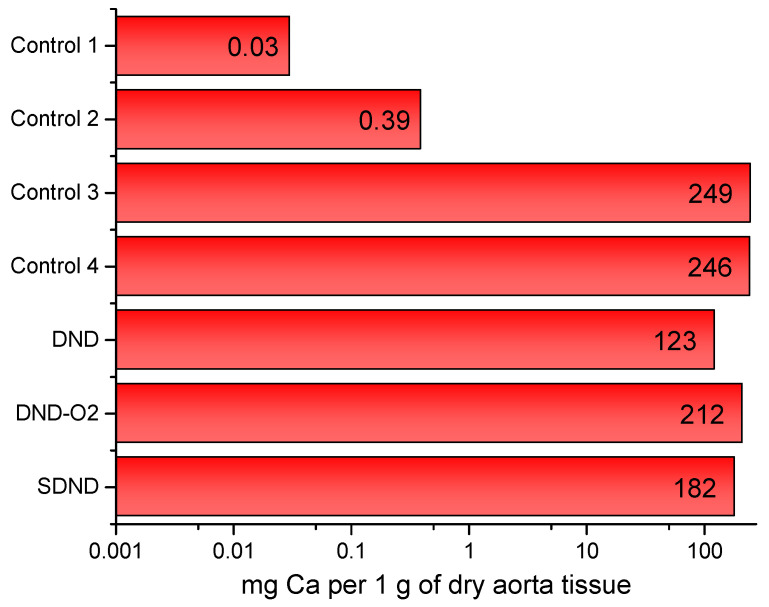
Calcium content in different types of nanodiamond-containing coatings. The solid line shows the median, error bars show a 5% acceptable range of values, and columns are the experimental data. Control 1 is the native pork aorta of the pig (far away from the allograft); Control 2 is the native pork aorta of the pig (near the allograft); Control 3 is the donor pork aorta devitalized by a hybrid approach; and Control 4 is the control inside the conduit (#4).

Both histological analysis and ICP-AES show that the calcification of the coated samples is commensurate with the control samples of another animal without nanodiamonds, which are also foreign materials to the body. Indeed, only the pig’s aorta was not calcified. This result confirms that nanodiamonds are not the source of the crystallization of insoluble calcium salts on the material’s surface. Moreover, the sample coated with DND shows a smaller calcification value than other materials (Figure 2). The reason is the positive surface charge of this type of nanodiamond. However, its toxicity is insufficient to prevent the growth of bacterial diseases [50].

Negatively charged nanodiamonds show a high ability to prevent the development of infection. Note that their efficiency can be significantly increased by the application of antibiotics that can be adsorbed on the developed highly functional surface of nanodiamonds [39]. SDNDs show a higher antibacterial efficiency than other nanodiamond types used in this study. The reason is the carboxylated surface of SDND nanodiamonds that contribute to the attachment to the bacterial cell wall surface [51]. Note that comparing the mechanical properties of the collagen materials coated with positively charged nanodiamonds with the materials containing smaller amounts of negatively charged nanodiamonds shows similar results in terms of increasing elasticity and rigidity [12]. Therefore, we can suggest that carboxylated nanodiamonds can be used as a separate coating for biomaterials for prosthesis production, while positively charged nanodiamonds require the adsorption of chemically bonded drugs to provide for the antimicrobial activity of the material. In both cases, the application of adsorbed antibiotics enhances the suppression of bacterial infections [39]. Note that nanodiamond-containing composites are prospective biomechanically active materials for bone mineralization and regeneration [40], while for soft aorta tissue modification, nanodiamonds have been used for the first time and have shown beneficial results, considering the well-being of the pig during the experiment.

## 4. Materials and Methods

### 4.1. Materials

A pork aorta was exposed to devitalization [48]. Detonation nanodiamonds were a product of PlasmaChem GmbH (Berlin, Germany). The following three types of nanodiamonds were used: DND, a nanodiamond powder with a positive charge when suspended in water; DND-O2, which was obtained by exposing DND to air annealing according to the procedure described in Reference [52]. Air annealing results in an increase in the negatively charged groups on the nanodiamonds’ surface and a total negative charge; and SDNDs, which are nanodiamonds supplied by the manufacturer as a suspension and have a negative surface charge. The nanodiamonds were tritium-labeled by the tritium thermal activation method [45].

### 4.2. Tritium Labeling of Nanodiamonds

Tritium-labeled nanodiamonds were obtained according to the procedure described in Reference [46]. Briefly, nanodiamonds were suspended in water up to a concentration of 0.376 g/L. A 0.8 mL portion of this suspension was equally distributed on the walls of the reaction flask and lyophilized. Then, the flask was connected to a special device designed for working with atomic hydrogen (tritium). The system was filled with tritium gas after evacuation. 

The reaction between nanodiamonds and tritium was performed for 10 s under the following conditions: a tungsten filament temperature of 2000 K, a tritium gas pressure of 1.5 Pa, and a temperature of nanodiamonds of 298 K. The residual gas was pumped out, the system was filled with a new portion of tritium gas, and the labeling was repeated. Then, the nanodiamonds were suspended in water and transferred into an Eppendorf tube. Initial radioactivity was measured in a GoldStar scintillator (Triskem, Brittany, France) using a liquid scintillation spectrometer RackBeta1215 (LKB Wallac, Turku, Finland).

To purify [^3^H]nanodiamonds from labile tritium, the suspension was stored at 4 °C for 24 h, followed by centrifugation and changing of the supernatant until the radioactivity of the supernatant reached the background level. After purification, the [^3^H]nanodiamonds were mixed with non-labeled material to prepare a suspension with a specific radioactivity of 9 mCi/g and 1 g/L in concentration.

### 4.3. Nanodiamond Coating Preparation and Animal Trial

The procedure of the [^3^H]nanodiamond suspension preparation is described in Section 4.2. Pieces of the aorta were placed in suspensions of [^3^H]nanodiamonds and stirred for 24 h, and then washed in water and stored in saline at 4 °C.

Control samples were dissolved in boiling nitric acid. The nanodiamonds’ content was separated by centrifugation and decantation. The sample was washed in water to reach a neutral pH. Then, a GoldStar scintillator (Triskem, Brittany, France) was added, and the radioactivity was measured using a RackBeta1215 liquid scintillation spectrometer (LKB Wallac, Finland). The number of nanodiamonds was determined as follows:

(1) $$N=\frac{I}{\varepsilon \;{\times \;a}_{sp}\times m\times 2.22\times 10^{9}}$$

where *I* is the rate count of tritium beta-radiation (counts per minute), *ε* is the registration efficiency, which was determined on the basis of the spectrum characteristics for each measurement, *a*_sp_ is the specific radioactivity of the nanodiamonds (mCi per g of nanodiamonds), *m* is the mass of the dry specimen of the aorta (g), and 2.22 × 10^9^ is the coefficient for recalculation decays per minute to mCi.

**Figure 3:**
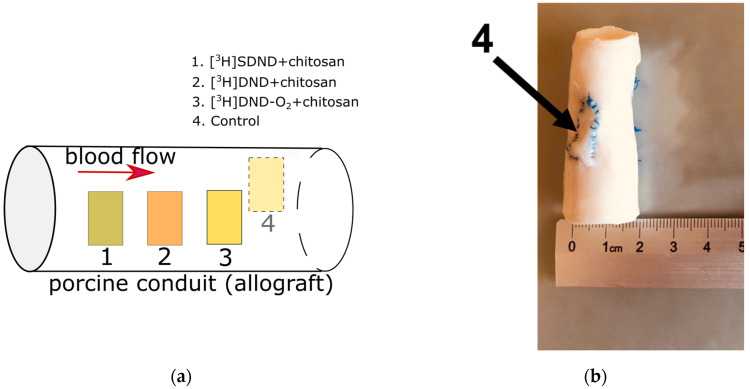

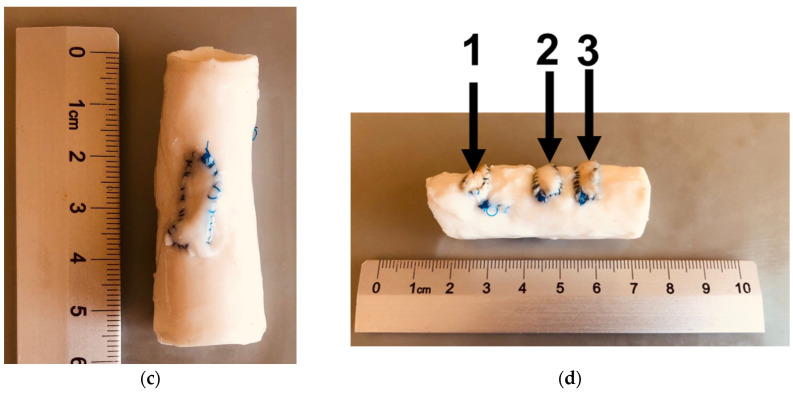
Scheme of the nanodiamond-containing samples in porcine conduit (**a**), (**b**–**d**) photo of the final allograft.

A chitosan layer was applied from the carbonic acid solution under high pressure [53]. Then, the pork aorta samples with the studied coatings were sewn inside a carrier, which is a donor pork aorta conduit (allograft) of 5.5 cm (2.17 in) in length and 2 cm (0.79 in) in diameter, which was devitalized (Figure 3).

The conduit was surgically stitched to the aorta of a 4-month-old Danish Landrace pig. After the surgery, the animal was healthy. The experiment involving a conduit in the animal’s aorta lasted for 4.5 months. After the experiment, the animal was euthanized; the allograft was extracted; the stitched samples with coatings, the control sample sewn inside the conduit, the carrier conduit, and the native aorta were tested for calcification and coating stability; and a histological analysis of the samples was performed.

**Figure 4:**
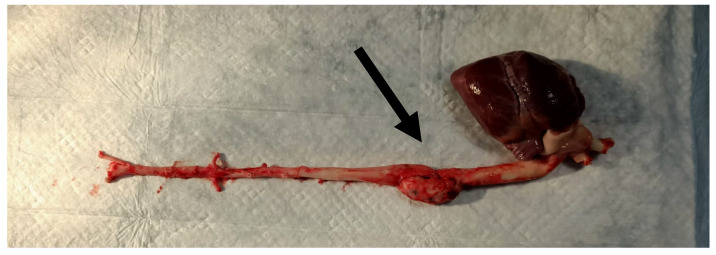
Construction of the sewn conduit after removal from the animal’s body. An arrow indicates the conduit.

Figure 4 depicts the construction of the sewn conduit after removal from the animal’s body.

The nanodiamond-containing samples were removed from the conduit and analyzed for nanodiamond and calcium content, and a histological analysis was performed.

### 4.4. Histological Analysis

Tissue samples from the experimental samples, as well as various sections of the conduit, including the native pig aorta, were placed in histological cassettes and fixed in a 10% buffered formalin solution for 24 h. The material was treated using a Leica ASP300S Fully Enclosed Tissue Processor (Leica Microsystems, Nussloch, Germany), followed by embedding it in paraffin. Slices of 3 to 5 μm thick were prepared using a Leica rotary microtome (Leica Microsystemssectio, Germany) and stained with hematoxylin and eosin on a Leica Autostainer XL (Leica Microsystems, Germany). A histological analysis was carried out using a Leica DMRB light microscope (Leica Microsystems, Germany) with a Leica DFC495 digital camera. Digital images were obtained using Leica Application Suite v 4.9.0 software (Leica Microsystems, Heerbrugg, Switzerland) with a 50× to 400× magnification.

### 4.5. Quantitative Analysis of the Coating for the Content of Nanodiamonds and Metals, Calcium in Particular 

The number of nanodiamonds in the coating was determined according to the procedure described in Section 4.3.

Calcium was traced by the ICP-AES method. After dissolving the sample in boiling nitric acid, the nanodiamonds were separated by centrifugation, while the solution was analyzed for calcium content by ICP-AES using an Agilent 720 spectrometer (Agilent, Mulgrave, VIC, Australia). A 10-fold diluted sample was diluted with deionized water. A series of ten calibration standards within the 0.1–100 ppm (mg/L) range were prepared from single-element CRMs (Certified Reference Materials, Inorganic Ventures, Christiansburg, VA, USA). The ICP-AES instruments were set up as follows: RF-power, gas flow rate, etc., according to the optimization experiments using a 5 ppm Mn and Ca stock solution. Wavelengths were chosen according to ISO 11885:2007 [54]. A spike recovery test was conducted for a trueness estimation. A 20 ppm Sc solution was used as an internal standard with real-time premixing to analyze the solution (not more than 5%) to increase the reproducibility of the results. Quality control was ensured through (1) analyzing two replicates of solutions and (2) 10 ppm 29-Element ICP Calibration/Quality Control Standard measurements.

## 5. Conclusions

The use of tritium-labeled nanodiamonds allows us to make the following conclusions regarding the improvement of a bioprosthetic valve coating. We can assume that detonation nanodiamonds make it possible to prepare relatively stable xenogenic material coatings for prosthesis production. The antimicrobial activity of carboxylated nanodiamonds substantiates their application as they are, while positively charged nanodiamonds require additional drugs to prevent the development of bacterial diseases. The application of such coatings does not lead to the excessive calcification of the material, suggesting their suitability for long-term use. Note that in the present research, we only analyzed a two-component material without antibiotic molecules adsorbed on nanodiamonds to prevent infection development. Our results allow us to choose SDND as the best type of nanodiamonds for heart valve prosthesis production, but not to discard other nanodiamonds that can be modified with antibiotics. In future research, we will analyze three-component coatings for histological changes and infection development. The limitation of the present study may be the duration of the experiment, which should be extended in the future.

Considering the rapid metabolism of the pig, and that the animal survived and appeared to cope very well with the allograft with the studied samples, we acknowledge the positive results related to the sufficient suppression of calcification and resistance to inflammation of the modified aortic tissue samples.

## 6. Ethical Conduct

All animal procedures were approved by the A.N. Bakulev National Medical Research Centre of Cardiovascular Surgery and conducted in accordance with ethical standards governing animal experiments in accordance with International and Russian regulatory documents (European Convention on Animal Care and Guide for the Care and Use of Laboratory Animals, as published by the US National Institutes of Health (NIH Publication № 85-23, revised 1996). Euroguide: On the Accommodation and Care of Animals Used for Experimental and Other Scientific Purposes. 2007. FELASA: Federation of European Laboratory Animal Science Associations, 25 Shaftesbury Avenue, London W1D 7EG, UK).

## 7. Patents

Patent RU2 711 544 C1 Biomaterial for making prostheses of heart valves and method of producing biomaterial. Accessed on 29 December 2018 Available online: https://patents.google.com/patent/RU2711544C1/ru.

## Figures and Tables

**Table 1:** Radioactivity of tritium-labeled nanodiamonds prepared by the tritium thermal activation method.

Type of Nanodiamonds	Radioactivity, mCi	
	Initial Radioactivity (Directly after the Reaction with Atomic Tritium)	After Purification from the Labile Tritium
DND	8.54	3.8
DND-O2	20.0	5.0
SDND	17.1	5.4

**Table 2:** The number of nanodiamonds in the coatings.

Type of Nanodiamonds	mg of Nanodiamonds per 1 g of Aorta Tissue	
	After Preparation	After Animal Exploitation
DND	2.00 ± 0.14	0.13 ± 0.05
DND-O2	3.00 ± 0.20	0.40 ± 0.10
SDND	5.50 ± 0.25	0.80 ± 0.20

**Table 3:** ICP-AES data on the aorta samples. The uncertainty of all results lies within 5%.

Sample Index	Metal Content, mg per 1 g of Dry Aorta Tissue				
	Ca	K	Mg	Na	Fe
Control sample of the native pork aorta of the pig (far away from the allograft)	0.03	0.004	0.01	1.66	9 × 10^−4^
Control sample of the native pork aorta of the pig (near the allograft)	0.39	0.23	0.13	22	0.02
Control sample inside the conduit (#4)	246	0.22	2.57	36	0.13
Control sample of the donor pork aorta devitalized by a hybrid approach	249	0.24	4.53	36	0.04
DND	123	0.30	1.13	36	0.13
DND-O2	212	0.22	3.11	39	0.11
SDND	182	0.28	1.86	42	0.15

## Data Availability

All data generated or analyzed during this study are included in this published article.
